# Dissociative Part-Dependent Resting-State Activity in Dissociative Identity Disorder: A Controlled fMRI Perfusion Study

**DOI:** 10.1371/journal.pone.0098795

**Published:** 2014-06-12

**Authors:** Yolanda R. Schlumpf, Antje A. T. S. Reinders, Ellert R. S. Nijenhuis, Roger Luechinger, Matthias J. P. van Osch, Lutz Jäncke

**Affiliations:** 1 Division of Neuropsychology, Institute of Psychology, University of Zurich, Zurich, Switzerland; 2 Department of Neuroscience, University Medical Center Groningen, and BCN Neuroimaging Center, University of Groningen, Groningen, The Netherlands; 3 Department of Psychosis Studies, Institute of Psychiatry, King's College London, London, United Kingdom; 4 Top Referent Trauma Center Mental Health Care Drenthe, Assen, The Netherlands; 5 Institute for Biomedical Engineering, University and ETH Zurich, Zurich, Switzerland; 6 Department of Radiology, C. J. Gorter Center for High-Field MRI, Leiden University Medical Center, Leiden, The Netherlands; 7 International Normal Aging and Plasticity Imaging Center, University of Zurich, Zurich, Switzerland; University of California, San Francisco, United States of America

## Abstract

**Background:**

In accordance with the Theory of Structural Dissociation of the Personality (TSDP), studies of dissociative identity disorder (DID) have documented that two prototypical dissociative subsystems of the personality, the “Emotional Part” (EP) and the “Apparently Normal Part” (ANP), have different biopsychosocial reactions to supraliminal and subliminal trauma-related cues and that these reactions cannot be mimicked by fantasy prone healthy controls nor by actors.

**Methods:**

Arterial spin labeling perfusion MRI was used to test the hypotheses that ANP and EP in DID have different perfusion patterns in response to rest instructions, and that perfusion is different in actors who were instructed to simulate ANP and EP. In a follow-up study, regional cerebral blood flow of DID patients was compared with the activation pattern of healthy non-simulating controls.

**Results:**

Compared to EP, ANP showed elevated perfusion in bilateral thalamus. Compared to ANP, EP had increased perfusion in the dorsomedial prefrontal cortex, primary somatosensory cortex, and motor-related areas. Perfusion patterns for simulated ANP and EP were different. Fitting their reported role-play strategies, the actors activated brain structures involved in visual mental imagery and empathizing feelings. The follow-up study demonstrated elevated perfusion in the left temporal lobe in DID patients, whereas non-simulating healthy controls had increased activity in areas which mediate the mental construction of past and future episodic events.

**Conclusion:**

DID involves dissociative part-dependent resting-state differences. Compared to ANP, EP activated brain structures involved in self-referencing and sensorimotor actions more. Actors had different perfusion patterns compared to genuine ANP and EP. Comparisons of neural activity for individuals with DID and non-DID simulating controls suggest that the resting-state features of ANP and EP in DID are not due to imagination. The findings are consistent with TSDP and inconsistent with the idea that DID is caused by suggestion, fantasy proneness, and role-playing.

## Introduction

Consistent clinical observations and retrospective findings indicate that dissociative identity disorder (DID) [1] is intimately related to severe traumatization, including emotional neglect [2]. This conclusion is supported by the results of prospective longitudinal research of dissociation [3], [4], [5]. Whereas most theories of DID include traumatization as one of the causal factors of the disorder [6], the sociocognitive model of DID involves the idea that the disorder is caused by suggestion, fantasy proneness, and role-playing [7], [8], [9], [10], [11], [12], [13]. However, studies showing that DID can be caused by these factors is lacking, and patients with DID are not particularly fantasy prone [14]. Also, mentally healthy women [15], high and low fantasy prone women [14], and actors [16] motivated and instructed to simulate two different prototypes of dissociative parts were unable to simulate the psychophysiological and neural activation patterns of these dissociative parts in women with DID.

Socioculture contexts affect any mental disorder [2], including dissociative disorders. However, there are no studies with DID patients showing that true positive cases are manufactured during (suggestive) psychotherapy, and that these patients' memories of childhood abuse are generally due to suggestion. Studies with DID patients have in fact disconfirmed the idea that the disorder results from the causes that the sociocognitive model of DID proposes [14], [15], [16]. For a discussion of this subject see Sar et al.: [17].

Several studies have compared psychobiological reactions of different dissociative parts in DID, but advances in the field critically depend on theoretical predictions with respect to the kind of biopsychosocial differences that exist among different types of dissociative subsystems or “parts” of the personality as a whole biopsychosocial system [18]. The Theory of Structural Dissociation of the Personality (TSDP) offers such hypotheses [2], [19], [20]. The two major prototypes of dissociative parts that TSDP distinguishes are metaphorically referred to as “Emotional Parts“ (EP) and “Apparently Normal Parts“ (ANP). As ANP, DID patients aim to fulfill functions in daily life, and in this context they try to mentally and behaviorally avoid traumatic memories and other trauma-related stimuli, commonly including EP. ANP, thus, has not or not sufficiently personified traumatic experiences and memories, can have a degree of amnesia regarding the traumatic past, and is to some degree depersonalized and bodily numbed. As EP, DID patients are fixated in traumatic memories, that is, in non-integrated sensorimotor and emotional reenactments of traumatizing events. There are two major subtypes of EP [19]. One subtype tends to engage in active mammalian defenses (e.g., freezing, flight, attachment cry) and strong emotions, such as intense fear, in reaction to actual or perceived threat. These reactions involve dominance of the sympathetic nervous system. As this subtype, DID patients are generally self-conscious, emotional (e.g., fearful), body-oriented, and hyperaroused. The other subtype of EP predominantly engages in passive mammalian defense (death feigning, also described as tonic immobility) to actual or perceived threat. This kind of defense would imply a degree of parasympathetically mediated hypoarousal, emotional numbing, and bodily anesthesia. Phenomenologically, ANP is predominantly characterized by dissociative symptoms that can be categorized as negative dissociative symptoms (functional losses), and EP (subtype active defense) by positive dissociative symptoms (e.g., re-enactments of traumatic experiences). Patients with DID commonly include more than one ANP and more than one EP. Some of their dissociative parts involve mixed features of ANP and EP.

TSDP is supported by clinical and empirical evidence. For example, in a Positron Emission Tomography (PET) study, DID patients listened as ANP and as EP (subtype active defense) to audiotaped descriptions of a neutral autobiographical memory that these dissociative parts had in common, as well as to a description of a traumatic memory that was only autobiographical for EP [21], [22]. ANP and EP (in Reinders et al. (2006) referred to as neutral identity state (NIS) and trauma-related identity state (TIS), respectively) had different psychophysiological and neural reaction patterns to the trauma script. In line with TSDP, as ANP, the patients had a brain activation pattern similar to patients with depersonalization disorder [23] and PTSD patients with negative dissociative symptoms to trauma-related stimuli [24], [25] (i.e., anterior cingulate cortex (ACC), somatosensory association area). ANP had highly similar reaction patterns to the neutral and the trauma script, which also indicates low emotional involvement in the trauma script. As EP (subtype active defense), patients were deeply emotionally and bodily engaged in this script. In contrast with ANP, EP had activation in many brain areas (i.e., insular cortex, amygdala, basal ganglia, cerebellum) also observed in PTSD patients who were confronted with a personalized trauma script and who reacted with positive symptoms such as subjective and physiological hyperarousal [26], [27], [28]. As EP but not as ANP, DID patients showed a significant increase in heart rate and blood pressure and a significant decrease in heart rate variability in reaction to the trauma script. In sum, EP was psychobiologically hyperaroused, and ANP was underengaged. The same paradigm was repeated with healthy matched controls [14]. Neither high nor low fantasy prone, mentally healthy women instructed and motivated to simulate ANP and EP had the psychophysiological and neural activation patterns of the genuine ANP and EP in DID patients. This finding contradicts the sociocognitive model of DID [7], [8], [9], [11], [13], [29].

With main effect and conjunction analyses [30], [31], Reinders et al. (2006) demonstrated that ANP and EP were associated with two different neural networks that are independent of the type of the memory script they listened to. The authors suggested that these networks might be involved in functioning as two different prototypes of dissociative parts. If this idea holds, ANP and EP should have different neural characteristics when instructed to rest, that is, to relax, close their eyes, and lay immobile on the back in the narrow enclosed MRI space with their head fixed, and without the distraction of a more specific task. According to TSDP, this assignment is emotionally challenging for DID patients most of whom have been chronically abused and emotionally neglected. The situation would be particularly demanding for them as EP. The experimental procedure could trigger trauma-related memories on which EP is fixated, and that ANP attempts to avoid.

The study of resting-state neural activity has recently become an important area of neuroimaging. Of special interest is the so called default mode network (DMN), a set of brain areas consisting of the medial prefrontal cortex (MPFC), posterior cingulate (PCC) in addition to midline parietal structures, lateral parietal regions, and medial and lateral temporal lobes [32], [33], [34]. The DMN is activated in response to rest instructions and is deactivated during the execution of goal-directed tasks [35], [36], [37], [38], [39], [40], [41]. Converging evidence suggests that the DMN is critical for general self-referential actions, such as autobiographical memory, self-reflection, self-awareness (i.e. introspection), and stimulus-independent thought [42], [43], [44], [45].

The main goal of the current study was to examine and compare brain perfusion patterns for ANP and EP (subtype active defense) in DID patients following rest instructions. Rest instructions, that is, instructions to relax and close the eyes, do not imply that the participants are actually resting. The term resting-state thus merely refers to the state that rest instructions elicit.

No study to date investigated so called resting-state perfusion differences in ANP and EP in DID. However, a Single-Photon Emission Computed Tomography (SPECT) study yielded bilateral orbitofrontal hypoperfusion and left lateral hyperperfusion during rest for a type of dissociative part in DID described as the “host” compared to healthy volunteers [46]. No significant perfusion differences were observed between the host, defined as the dissociative part of the personality that is most of the time present during a usual day [47], and an “alter”, a different type of dissociative part. We suspect that in most cases, the “host” was an ANP, but it is unclear if the “alter” involved a second ANP, an EP prone to engage in active or passive mammallian defense, or an ANP/EP mixture. If the “host” and the “alter” involved two ANPs rather than an ANP and an EP (subtype active defense), this might explain why Sar et al. [46] did not find different patterns of brain activity for the tested dissociative parts. A different but not incompatible possibility is that SPECT is insufficiently sensitive to measure perfusion differences in response to rest instructions. In the current study, we used more sensitive arterial spin labeling (ASL), which generates PET-like images without the need of a radioactive tracer [48]. ASL provides a quantitative CBF measurement, and is therefore particularly useful in the investigation of individual differences in brain metabolism [49].

In addition to the comparison of brain perfusion patterns for ANP and EP in DID patients, the current study also investigated neural activation patterns for ANP and EP in healthy simulating controls (SIM) in order to test the idea that DID involves suggestion and role-playing rather than a trauma-related condition [9], [29]. According to TSDP, simulating an ANP and EP and being a genuine ANP and EP constitute different mental states. It can be expected that in contrast to DID simulating controls, DID patients engage as ANP and EP in self-referential actions following rest instructions.

Considerations based on TSDP and the mentioned empirical foundation lead us to hypothesize that (i) there are perfusion differences for DID patients and DID simulating controls. In particular, we predicted that (ia) compared to the actors, DID patients show relatively higher activation in areas which commonly exhibit increased neural activity following rest instructions (default mode activity), i.e. MPFC, PCC/precuneus, medial and lateral parietal areas, and medial and lateral temporal regions. Looking at the comparison from the other side, we hypothesized that (ib) actors compared to DID patients elicit less default mode activity because simulating an ANP and EP involves a goal-directed task. We furthermore hypothesized that in response to the described rest instructions, (ii) ANP and EP in DID have different patterns of brain perfusion and that (iii) comparisons of ANP and EP simulating controls yield different neural reactivity patterns than comparisons of ANP and EP in DID patients.

## Methods

### Participants

Fifteen female DID patients were included in the current study, which was part of a larger study in DID patients and DID simulating controls [16]. We enrolled Swiss and German patients, who were recruited from private practitioners of psychiatry and psychotherapy and psychiatric outpatient departments. All participants fulfilled the diagnostic criteria of DID according to DSM-IV [1]. For the sake of the study, the clinical diagnoses were independently checked by experts in dissociative disorders using the German version of the Structured Clinical Interview for DSM-IV Dissociative Disorders (SCID-D) [50], the (SKID-D) [51]. The therapy of the participating patients had to have progressed to a treatment phase involving integration-focused exposure to traumatic memories [2], [52]. Exclusion criteria were comorbid psychosis, drug abuse or addiction, antisocial or histrionic personality disorder, and a neurological or organic brain disease. Thirteen patients were medicated at the time of the measurement, predominantly with antidepressant medication. Two patients were free of medication.

The DID simulating control group (SIM) consisted of 15 female actors, who were instructed and motivated to simulate ANP and EP during the resting-state experiment. They were carefully informed about the characteristics of ANP and EP using written information on TSDP. They also watched a video showing a DID patient, who alternates between ANP and EP. The actors were instructed to create an ANP and EP using a list of properties (e.g., name, sex, age). Of particular importance was that ANP should be a dissociative part without personalized memories of traumatizing events and EP a dissociative part with personalized traumatic memories. The actors were requested to practice simulating ANP and EP as often as they deemed necessary to effectively simulate ANP and EP, but at least three times before the MRI measurement.

There were no significant differences between the DID and SIM group in age (DID: M = 43.3 years, SD = 9.1; SIM: M = 43.2 years, SD = 10.4; t(28) = 0.019, p>0.05) and educational level (DID: M = 4.1, SD = 1.5; SIM: M = 4.7, SD = 1.2; t(26.099) = −1.341, p>0.05; the educational level was assessed by a 7-point Likert scale based on the common European educational system). To ensure that none of the actors had a PTSD and/or major depression, the controls completed the German version of the Posttraumatic Diagnostic Scale (PDS) [53] and the Beck Depression Inventory II (BDI-II) [54].

Each subject was informed about risks and inconveniences associated with the experiment. All subjects gave written informed consent. The local ethics committee (cantonal ethical commission of Zurich) approved the study in compliance with the Helsinki Declaration.

### Experimental Design and Procedure

The subjects were instructed to relax with their eyes closed and to stay motionless during the fMRI measurement (e.g., [34]). All participants of the DID and SIM group were first tested as ANP and next as EP, because starting with the less anxious dissociative part might be less demanding for DID patients. The switch between the different dissociative parts of the personality took place outside the scanner, if needed with minimal guidance from the research clinician. Because each dissociative part includes his/her own conception of self [55], and because the participating patients had developed the ability in treatment to switch on request between the participating ANP and EP, it was possible to ask these dissociative parts to “come forward” or “step back” during the experiment. For example, when the EP was to be tested, addressing this part by her name, she was invited to “come forward”. If she had brought a personal object with her (e.g., a cuddle toy), the patient was asked to hold this object in her hands to support the switching process. Meanwhile, addressing the ANP by her name, she was asked to “step back”. To check for inadvertent switches to and co-activation of one or more different dissociative part than the intended ANP or EP, we asked the participants after each run what part had been present during the measurement. One ANP and two EP runs had to be repeated due to a switch to and/or a co-activation of an unintended dissociative part.

### Image Acquisition and Data Preprocessing

The data of the DID and SIM group were obtained at the University Hospital of Zurich with a 3-T Philips Achieva whole-body magnetic resonance imaging equipped with an eight-channel Philips SENSE head coil. Resting regional cerebral perfusion (rCBF) images were acquired with a pseudo-continuous ASL (p-CASL) sequence with background suppression (saturation of the imaging slice preceding the labeling and inversion pulses 1680 ms and 2760 ms after the saturation pulse) and a single shot echo-planar imaging (EPI) readout (TR/TE = 4180/12 ms, SENSE factor 2.5) [56]. The duration of the labeling was 1650 ms and the image was acquired after a delay of 1525 ms. The sequence consisted of 23 slices of 6 mm slice thickness acquired in ascending order with a 3×3 mm^2^ in-plane resolution. During a single run, 35 pairs of control/label image volumes were measured over a total scan time of 5 minutes. An additional M0 image was acquired for measurement of the magnetization of arterial blood (same sequence as ASL without labeling or background suppression, TR = 10 s). A 3D MPRAGE T1-weighted anatomical scan was acquired for anatomical reference and post-processing.

The data of three actors and one patient were excluded due to huge movement artefacts and low signal quality. One patient had fallen asleep during the ANP and had switched several times during the EP run. One patient was not able to undergo the MRI measurement, and the data of one patient were lost due to a storage failure at the MRI center. The final brain imaging statistical analysis was performed with data of 11 participants in the DID group and 12 in the SIM group.

Resting-state rCBF maps were calculated using in-house programmed MATLAB scripts performing a simple pair-wise subtraction of control and label images [56]. Further analyses were performed with the statistical parametric mapping software SPM8 (http://www.fil.ion.ucl.ac.uk/spm). rCBF maps were normalized to the EPI template [57], which transformed them into MNI space (new voxel size = 2×2×2 mm^3^). The normalized rCBF maps were spatially smoothed with an 8-mm full width at half-maximum (FWHM) Gaussian kernel.

The preprocessed data were analyzed using a flexible factorial design that consisted of two independent variables resulting in a 2×2 ANOVA with repeated measures on the second factor: Group (two levels: DID/SIM), Type of dissociative part of the personality (two levels: ANP/EP). The second factor will be referred to as *Type* in the rest of the article. In order to correct for biological variation in total cerebral blood flow, the mean gray matter (GM) CBF was included in the analysis as a covariate of no interest. The mean GM signal per subject was calculated over a GM mask obtained from the segmentation of the 3D T1 image by thresholding the GM probability images at 0.5. Only the GM signal was taken into account, as a previous study revealed that GM perfusion showed most variability between sessions [58].

We performed a whole-brain voxel-wise analysis. The study design allows the calculation of various effects, i.e. main effect of Group, main effect of Type, and interaction effect Group by Type. An uncorrected statistical threshold (i.e., voxel level of significance uncorrected (unc.) for multiple testing) of p<0.005 was set for the main effects and interaction effect. The minimum cluster-size was set at 10 voxels.

Our main hypotheses were tested using one-sided t-tests. The participants were measured as ANP and EP in the patient group (DIDanp/DIDep) and in the control group (SIManp/SIMep). Group differences between the patients (DID) and actors (SIM) were assessed with a two-sample t-test based on the mean perfusion map of ANP and EP of every single participant (DID-SIM). Two planned comparisons consisting of Type effects between groups (DIDanp-SIManp; DIDep-SIMep) and two planned comparisons consisting of Type effects within groups (DIDanp-DIDep; SIManp-SIMep) were performed. All five t-tests were one-sided, thus were performed twice to assess positive rCBF differences in one and in the inverse contrast. To keep statistical thresholding of a priori defined regions conservative enough, we used a Bonferroni correction for these subsequent tests (p<0.005/5 = p<0.001). Thus, all t-tests were fixed to a p<0.005 and corrected for the number of tests. A similar approach has been used in Schlumpf et al. (2013).

An explicit binary mask provided by FSL (http://www.fmrib.ox.ac.uk/fsl) was applied at the level of the statistical interference to remove extracranial voxels. The mask was normalized to MNI space and had the same dimension and voxel size as the rCBF maps. Only the most significant finding of a brain area and first peak of a cluster are reported in the **Table 1** to **4**. The cluster locations were labeled using the Harvard-Oxford cortical and subcortical structural atlases [59] and by visual inspection on a high-resolution T1-weighted image in FSL. Subregions in the cingulate cortex were named according to Vogt's division based on cytoarchitectonic characteristics [60]. The results are restricted to activations in the GM, as white matter perfusion measurements are still challenging with ASL [56].

**Table 1:** Main effect of Group, main effect of Type (ANP/EP), and interaction effect on resting-state regional cerebral blood flow (rCBF).

			MNI coordinates^a^				
	Brain area	Side	*x*	*y*	*z*	k*E*	*F* value
Main effect of Group	Superior temporal gyrus (temporal pole)	L	−54	2	−24	154	26.81
	Precuneus	L	−10	−54	56	171	23.17
	Angular gyrus	R	48	−48	28	318	21.81
	Middle frontal gyrus	R	36	14	32	46	19.53
	Superior frontal gyrus	R	16	40	42	64	17.53
	Occipital fusiform gyrus	L	−36	−74	−16	60	16.78
	Occipital pole (extrastriate cortex)	L	−28	−94	−24	13	14.56
	Temporal occipital fusiform cortex	L	−40	−52	−18	56	13.59
	Inferior frontal gyrus (pars triangularis)	L	−54	32	6	39	13.45
	Lateral occipital cortex (inferior division)	L	−46	−82	10	24	13.44
	Thalamus	R	18	−20	14	31	12.49
	Hippocampus	R	32	−12	−26	19	12.20
Main effect of Type	Pre-SMA	R	10	10	66	193	17.61
	Postcentral gyrus (primary somatosensory cortex)	R	56	−12	36	89	16.95
	DMPFC	R	14	64	32	100	15.63
	Superior parietal lobe	R	38	−56	62	53	15.50
	Inferior temporal gyrus (temporal pole)	R	44	2	−50	25	13.86
	Insula (anterior)	R	30	14	−14	47	13.82
	Thalamus	R	10	−18	12	29	12.63
	PgACC	R	2	42	0	65	11.39
	Inferior temporal gyrus (temporal pole)	L	−44	−2	−46	26	11.24
	Occipital pole (extrastriate cortex)	L	−30	−94	0	34	11.09
	Thalamus	L	−12	−28	12	19	10.89
	SMA	R	2	−22	64	14	10.43
	MPFC	R	10	66	−8	17	10.24
Interaction effect	Inferior temporal gyrus	R	52	−64	−12	52	17.69
	Thalamus	R	8	−16	4	122	17.48
	Postcentral gyrus (primary somatosensory cortex)	R	60	−16	36	131	16.67
	Putamen	R	24	6	12	97	16.17
	SgACC	R	6	30	−6	48	14.50
	Central operculum	R	46	−2	10	337	14.40
	Amcc	L	−6	24	20	215	13.28
	Parietal operculum	L	−48	−28	18	160	13.11
	Frontal operculum	R	38	14	8	29	11.63
	pMCC	L	−8	−20	36	59	11.51
	Middle temporal gyrus (temporal pole)	L	−48	16	−36	15	11.27
	Thalamus	R	10	−22	18	50	11.20
	Insula (anterior)	L	−30	8	−16	22	10.89
	Insula (posterior)	R	30	10	−16	14	10.38
	Pre-SMA	L	−6	12	50	10	10.38
	Superior frontal gyrus	R	6	20	44	12	10.19
	sgACC	L	−4	14	−4	35	9.78

R/L, left or right hemisphere; k*E*, clustersize in voxels (one voxel is 2×2×2 mm); Pre-SMA, pre-supplementary motor area; DMPFC, dorsomedial prefrontal cortex; pgACC, perigenual anterior cingulate cortex; SMA, supplementary motor area; MPFC, medial prefrontal cortex; sgACC, subgenual anterior cingulate cortex; aMCC, anterior midcingulate cortex, pMCC, posterior midcingulate cortex;

a MNI coordinates (in mm) refer to the maximum of signal change in each region.

**Table 2:** Group differences in resting-state regional cerebral blood flow (rCBF).

			MNI coordinates^a^				
	Brain area	Side	*x*	*y*	*z*	k*E*	*T* value
DID-SIM	Middle temporal gyrus (temporal pole)	L	−54	2	−24	102	5.18
	Precuneus	L	−10	−54	56	87	4.81
	Angular gyrus	R	48	−48	28	99	4.67
	DMPFC	R	16	40	42	17	4.19
	Superior frontal gyrus	L	−20	14	46	25	4.02
SIM-DID	Middle frontal gyrus	R	36	14	32	24	4.42
	Occipital fusiform gyrus	L	−36	−74	−16	25	4.10

R/L, left or right hemisphere; k*E*, cluster-size in voxels (one voxel is 2×2×2 mm); n.s., not significant; DID, patient group; SIM, DID simulating control group; DMPFC, dorsomedial prefrontal cortex.

a MNI coordinates (in mm) refer to the maximum of signal change in each region.

**Table 3:** Type (ANP/EP) effects between groups on resting-state regional cerebral blood flow (rCBF).

			MNI coordinates^a^				
	Brain area	Side	*x*	*y*	*z*	k*E*	*T* value
DIDanp - SIManp	Angular gyrus	R	46	−48	28	240	5.26
	Middle temporal gyrus (temporal pole)	L	−56	4	−26	87	4.80
	dPCC/Precuneus	R	8	−54	32	12	3.75
	Precuneus	L	−12	−52	58	25	3.63
SIManp - DIDanp	Middle frontal gyrus	R	34	14	30	39	4.02
DIDep - SIMep	Precuneus	L	−14	−52	56	116	4.47
	DMPFC	R	16	40	40	51	4.43
	Angular gyrus	R	46	−48	28	37	4.05
	SMA	L	−14	−8	64	19	3.94
	Superior frontal gyrus	L	−20	16	46	34	3.81
	Middle temporal gyrus (temporal pole)	L	−58	4	−26	23	3.63
SIMep - DIDep	Thalamus	R	18	−22	16	60	3.94
	Middle frontal gyrus	R	36	14	32	16	3.93
	Superior parietal lobe	L	−56	−48	54	11	3.76
	Hippocampus	R	32	−14	−26	27	3.67
	Occipital fusiform gyrus	L	−38	−74	−14	20	3.66
	Lateral occipital cortex (middle division)	L	−38	−76	10	14	3.53
	Temporal occipital fusiform gyrus	L	−38	−52	−16	10	3.50

R/L, left or right hemisphere; k*E*, cluster-size in voxels (one voxel is 2×2×2 mm); DIDanp, ANP DID group; DIDep, EP DID group; SIManp, ANP control group; SIMep, EP control group; dPCC, dorsal posterior cingulate cortex; DMPFC, dorsomedial prefrontal cortex; SMA, supplementary motor area.

a MNI coordinates (in mm) refer to the maximum of signal change in each region.

**Table 4:** Type (ANP/EP) effects within groups on resting-state regional cerebral blood flow (rCBF).

			MNI coordinates^a^				
	Brain area	Side	*x*	*y*	*Z*	k*E*	*T* value
DIDanp - DIDep	Thalamus	R	10	−22	16	42	3.95
	Thalamus	L	−6	−22	16	13	3.52
DIDep - DIDanp	Postcentral gyrus (primary somatosensory cortex)	R	58	−14	36	139	5.18
	Pre-SMA	R	12	8	66	141	4.48
	Middle temporal gyrus (temporal pole)	R	26	24	−34	12	4.18
	Precentral gyrus (primary motor cortex)	R	38	−14	36	74	4.16
	DMPFC	R	14	48	34	41	4.06
	Superior parietal lobe	R	36	−56	64	26	3.71
	Precentral gyrus (premotor cortex)	R	16	−12	66	15	3.61
SIManp - SIMep	Thalamus	R	8	−16	8	82	4.59
	Occipital pole (extrastriate cortex)	L	−10	−98	16	29	4.10
	Thalamus	L	−4	−2	2	40	3.90
	Occipital pole (extrastriate cortex)	R	24	−94	14	23	3.57
SIMep – SIManp	Insula (anterior)	R	30	12	−16	102	4.87
	Frontal operculum	R	38	14	8	127	4.01
	Pre-SMA	L	−8	12	52	25	3.92
	Inferior temporal gyrus (temporal pole)	R	44	4	−48	20	3.83
	Inferior frontal gyrus (pars triangularis)	L	−46	30	0	27	3.78
	Putamen	R	26	4	12	63	3.76
	Middle temporal gyrus (temporal pole)	L	−48	16	−34	19	3.66
	Central operculum	L	−34	6	14	38	3.65
	pgACC	R	8	36	−8	10	3.60
	OFC	L	−26	8	−16	14	3.55
	Pallidum	R	14	−4	−6	10	3.50
	Pallidum	L	−20	−6	−6	12	3.43

R/L, left or right hemisphere; k*E*, cluster-size in voxels (one voxel is 2×2×2 mm); DIDanp, ANP DID group; DIDep, EP DID group; SIManp, ANP control group; SIMep, EP control group; Pre-SMA, pre-supplementary motor area; DMPFC, dorsomedial prefrontal cortex; pgACC, pregenual anterior cingulate cortex; OFC, orbitofrontal cortex.

a MNI coordinates (in mm) refer to the maximum of signal change in each region.

## Results

### Repeated Measures ANOVA

Significant rCBF differences for the Group main effect, independent of Type, and for the Type main effect, independent of Group, were found. In addition, significant perfusion differences were observed due to an interaction effect between Group and Type (see **Table 1**).

### Group differences

Group differences are given in **Table 2**. In line with our first hypothesis, we found positive perfusion differences in the patient group compared to the actors (DID-SIM) and in the actors compared to the patient group (SIM-DID).

DID showed higher perfusion than SIM in the temporal pole of the middle temporal gyrus, in the precuneus, angular gyrus, and in the dorsomedial prefrontal cortex (DMPFC). In SIM compared to DID, we observed increased perfusion in the middle frontal gyrus and the occipital fusiform gyrus.

### Planned comparisons

Between-group comparisons of Type (i.e., two different types of dissociative parts of the personality, ANP/EP) are listed in **Table 3**. Type comparisons within groups are given in **Table 4**. We found significant rCBF differences in all eight planned comparisons.

#### Between-group Type comparisons

Significant rCBF changes for both ANP and EP between the groups are in accordance with our first hypothesis.

Compared to SIManp, DIDanp was associated with more activation in the angular gyrus, temporal pole of the middle temporal gyrus, dorsal posterior cingulate cortex (dPCC), and precuneus (DIDanp-SIManp). In the inverse contrast (SIManp-DIDanp), we found a higher perfusion in the middle frontal gyrus. Increased activation in the precuneus, DMPFC, angular gyrus, and temporal pole of the middle temporal gyrus was found in the contrast DIDep-SIMep. In SIMep compared to DIDep (SIMep-DIDep), we observed increased perfusion in the thalamus and in several occipital-temporal regions (i.e., occipital fusiform gyrus, lateral occipital cortex, temporal occipital fusiform cortex.

#### Within-group Type comparisons

The second hypothesis that DIDanp and DIDep differ in resting-state perfusion could not be rejected given significant rCBF differences in DIDanp-DIDep and DIDep-DIDanp. In line with our third hypothesis, comparisons of SIManp and SIMep yielded different neural reactivity patterns than comparisons of DIDanp and DIDep.

**Figure 1:**
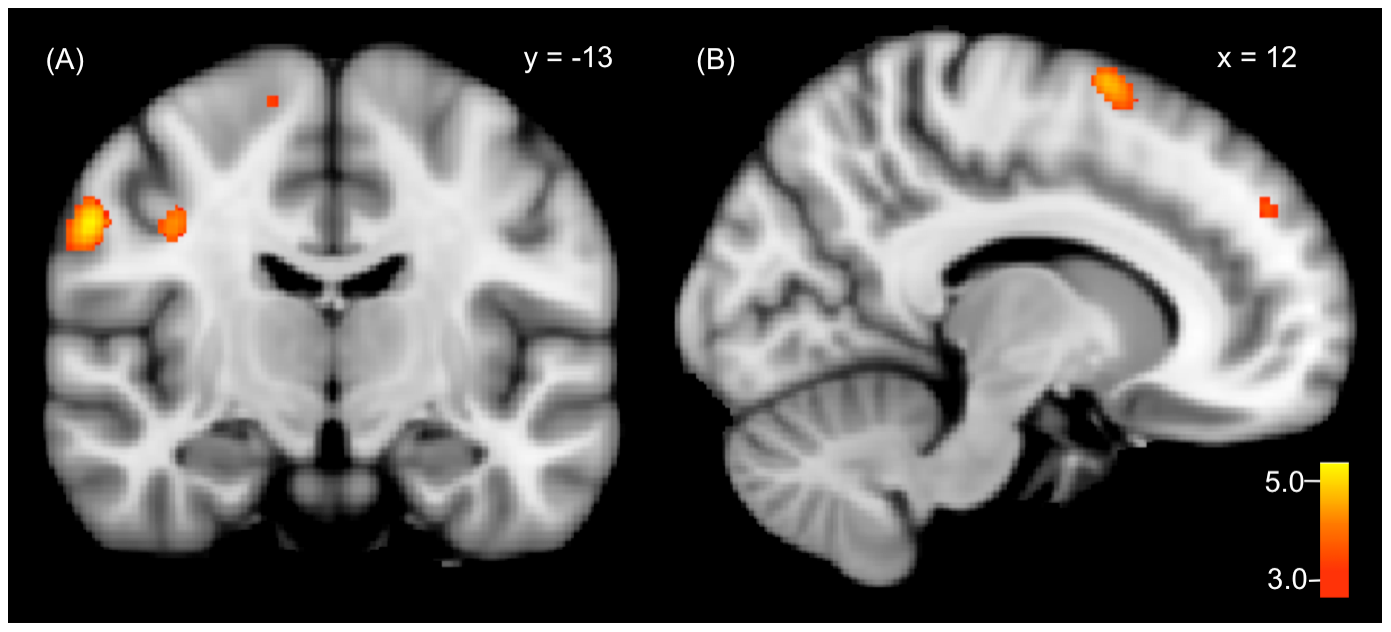
Significant rCBF increases in genuine EP (DIDep) compared to genuine ANP (DIDanp) in (A) the primary somatosensory cortex, primary motor cortex, premotor cortex and in (B) the pre-SMA and DMPFC.

DIDanp had more perfusion in the bilateral thalamus than DIDep (DIDanp-DIDep). The inverse contrast (DIDep-DIDanp) showed increased perfusion in the primary somatosensory cortex and in several motor-related brain areas including the primary motor cortex and higher-order motor areas (i.e., pre-supplementary motor area (pre-SMA), premotor cortex). DMPFC hyperperfusion was also observed (**Figure 1**).

Comparing SIManp to SIMep (SIManp-SIMep) revealed higher brain activation in the right thalamus and in the extrastriate cortex. In the inverse contrast (SIMep-SIManp), we observed a higher perfusion in insular-opercular regions (anterior insula, frontal operculum) and in inferior frontal areas (pars triangularis of the inferior frontal gyrus, orbitofrontal cortex (OFC)).

## Follow-Up Study

### Methods

Simulating a resting DID patient is a paradoxical situation because resting and simulating are opposite actions. Resting instructions invite relaxed, internally directed attention, whereas instructions to simulate a dissociative part elicit effortful goal-directed action. This paradox did not exist in a follow-up study in which the mentally healthy control group (NS) only received resting instructions. The NS group consisted of 15 female participants. There were no significant differences between the DID and NS group in age (DID: M = 43.3 years, SD = 9.1; NS: M = 38.20 years, SD = 10.3; t(28) = 1.428, p>0.05) and educational level (DID: M = 4.1, SD = 1.5; NS: M = 5.1 years, SD = 1.4; t(28) = −2.018, p>0.05).

Atypical default mode activity has been observed in a number of mental disorders [61]. Only few resting-state imaging studies of PTSD have been performed [62], [63], [64], [65], [66]. The findings are heterogenous and are not restricted to the default mode activity, even though abnormal resting-state activity in some areas of the DMN was observed in PTSD patients compared to healthy controls [62], [66]. A resting-state study of DID patients is lacking to date. As this disorder includes significant disturbances in self-referential actions, it can be expected that DID patients and non-simulating healthy participants differ in their default mode activity.

For the follow-up study, the data of the NS group were obtained on the same scanner which has been used for the DID and SIM group, but after an upgrade from the Achieva to the Ingenia system (different MRI system). The involved system changes are described in the Supporting Information S1 (see **Text S1 in Information S1**). The data set of 15 participants of the NS group were compared with the data set of the DID group (n = 11). For the pre- and postprocessing of the NS data set, the same strategy was used as for the other two groups. The following three two-sample t-tests were performed: DID-NS, DIDanp-NS, DIDep-NS. All three t-tests were one-sided, and were performed twice to assess positive rCBF differences in one and in the inverse contrast. Bonferroni corrected p-values were used (p<0.005/3 = p<0.00167) and cluster-size threshold was set at 10 voxels.

## Results

DID and NS showed a different resting-state activity pattern within the DMN (see **Table 5**). For DID compared to NS (DID-NS), we observed increased activity within the left temporal gyrus (i.e., posterior and polar part of the superior temporal gyrus, middle temporal gyrus, and inferior temporal gyrus), OFC, and DMPFC. Elevated rCBC in left temporal regions were also present in DIDanp compared to NS (DIDanp-NS) and in DIDep compared to NS (DIDep-NS). For the latter contrast, we also found elevated perfusion in the OFC and DMPFC.

**Table 5:** Resting-state regional cerebral blood flow (rCBF) differences between DID patients and non-simulating controls.

			MNI coordinates^a^				
	Brain area	Side	*x*	*y*	*Z*	k*E*	*T* value
DID-NS	Middle temporal gyrus (temporal pole)	L	−62	6	−20	187	5.82
	Inferior temporal gyrus	L	−38	0	−38	172	5.37
	Superior frontal gyrus	L	−26	42	50	41	4.75
	OFC	L	−6	26	−18	339	4.64
	Middle temporal gyrus	L	−70	−30	−10	12	4.16
	DMPFC	R	16	64	34	10	4.02
NS-DID	Supramarginal gyrus	L	−58	−42	44	286	6.65
	Occipital pole (extrastriate cortex)	L	−26	−98	2	515	*5.32
	MPFC (frontopolar cortex)	R	4	54	−10	150	4.58
	Angular gyrus	L	−52	−60	30	122	4.44
	Supramarginal gyrus	R	62	−36	44	129	4.43
	Lateral occipital cortex (middle division)	L	−40	−84	12	29	4.43
	Superior temporal gyrus	R	58	0	2	18	4.31
	Frontal pole	R	46	42	−2	32	4.22
	Lateral occipital cortex (superior division)	L	−24	−76	32	39	4.15
	Lateral occipital cortex (middle division)	R	48	−86	4	40	4.06
	Inferior frontal gyrus (pars opercularis)	L	−50	18	30	20	4.05
	Insula (posterior)	L	−34	−22	14	21	3.96
	Inferior temporal gyrus	R	32	−32	−8	13	3.71
	Hippocampus	R	32	−32	−8	13	3.71
DIDanp-NS	Middle temporal gyrus (temporal pole)	L	−62	6	−22	92	5.13
	Superior frontal gyrus	L	−26	42	50	53	4.69
	Superior temporal gyrus	L	−70	−30	10	17	4.12
	Inferior temporal gyrus (temporal pole)	L	−36	2	−42	58	4.08
	Middle temporal gyrus	L	−58	−40	−8	11	3.89
	Middle frontal gyrus	L	−46	52	14	31	3.74
NS-DIDanp	Supramarginal gyrus	L	−58	−40	44	203	5.84
	Middle frontal gyrus	R	30	−2	48	118	5.16
	Occipital pole (extrastriate cortex)	L	−26	−98	2	372	*4.90
	MPFC (frontopolar cortex)	R	4	52	−8	113	4.88
	Superior temporal gyrus	R	58	0	2	25	4.50
	Supramarginal gyrus	R	64	−28	34	92	4.43
	Lateral occipital cortex (superior division)	L	−24	−76	30	35	4.20
	Lateral occipital cortex (inferior division)	R	48	−86	4	63	4.17
	Middle frontal gyrus	L	−48	18	28	21	4.12
	Frontal pole	R	44	44	−2	19	4.04
	Angular gyrus	L	−42	−70	46	45	4.01
	Inferior temporal gyrus	R	46	−40	−24	10	3.76
	Putamen	R	24	8	−4	12	3.73
	Hippocampus	R	30	−34	−8	12	3.67
	Occipital pole (extrastriate cortex)	R	22	−98	12	19	3.58
DIDep-NS	Middle temporal gyrus (temporal pole)	L	−66	0	−18	286	6.65
	OFC	R	6	24	−26	256	4.98
	Superior frontal gyrus	L	−26	42	50	36	4.65
	DMPFC	R	16	64	34	18	4.62
	Superior temporal gyrus	L	−70	−30	−10	21	4.45
	Inferior temporal gyrus	L	−62	−38	−24	36	4.32
	Middle frontal gyrus	R	54	30	36	26	3.86
	Precentral gyrus	L	−2	−26	58	10	3.69
	Superior frontal gyrus	R	20	2	58	21	3.68
NS-DIDep	Supramarginal gyrus	L	−58	−42	46	301	6.69
	Lateral occipital cortex (middle division)	L	−40	−84	12	657	*5.49
	Angular gyrus	L	−52	−60	30	274	5.13
	Insula (posterior)	L	−34	−22	14	54	4.45
	Supramarginal gyrus	R	62	−36	44	88	4.35
	Inferior frontal gyrus (pars opercularis)	L	−50	18	32	25	4.27
	MPFC (frontopolar cortex)	R	4	56	−10	140	4.13
	Frontal pole	R	46	42	−2	23	4.05
	Inferior temporal gyrus	R	46	−40	−22	15	4.02
	Lateral occipital cortex (inferior division)	R	48	−86	4	32	3.90
	Lateral occipital cortex (superior division)	L	−30	−60	46	31	3.84
	Hippocampus	R	32	−32	−8	19	3.72

R/L, left or right hemisphere; k*E*, cluster-size in voxels (one voxel is 2×2×2 mm); DID, patient group; NS, non-simulating control group; DIDanp, ANP DID group; DIDep, EP DID group; OFC, orbitofrontal cortex; DMPFC, dorsomedial prefrontal cortex; MPFC, medial prefrontal cortex.

a MNI coordinates (in mm) refer to the maximum of signal change in each region.

*corrected for multiple comparisons using cluster-level statistics, *p*≤0.05.

NS compared to DID (NS-DID) had increased perfusion in posterior parietal regions (i.e., supramarginal gyrus, angular gyrus), in several occipital areas (i.e., occipital pole, lateral occipital cortex), in the frontal polar cortex, in the right superior and inferior temporal gyrus, and in the hippocampus. Increased rCBF in posterior parietal regions, occipital areas, frontal polar regions, and hippocampus were also present for NS compared to DIDanp (NS-DIDanp) and for NS compared to DIDep (NS-DIDep).

## Discussion

This is the first fMRI perfusion study measuring brain perfusion following rest instructions in DID patients. As hypothesized, we found differences between DID patients and DID simulating actors, as well as between two different prototypes of dissociative parts of the personality (ANP and EP) in DID patients. The follow-up study demonstrated different perfusion patterns for DID and controls who did not simulate ANP and EP.

Compared to actors, DID patients showed higher resting-state metabolism in several areas belonging to the DMN (i.e, temporal pole of the middle temporal gyrus, precuneus, angular gyrus, and DMPFC) [32]. The default mode activity of DID is in line with our first hypothesis and suggests that DID patients were more involved in attending to their self-states when instructed to rest than actors. In the inverse contrast (SIM-DID), we found more perfusion in the middle frontal gyrus and occipital fusiform gyrus. The DMN is also known as „task-negative“ network [37]. Whereas it shows attenuated levels of neural activity at rest and during self-referential processes [33], [34], [42], [43], [44], [45], this network exhibits activity decreases across many goal-directed tasks [35], [36], [37], [38], [39], [40], [41]. The fact that enacting ANP and EP involves a goal-directed task can explain the relatively lower default mode activity for DID simulating controls compared to DID patients.

The between-group Type effects fit to these interpretations. Of special interest is the increased activity in the precuneus and angular gyrus for ANP and EP in DID patients when contrasted with the corresponding simulated ANP and EP (i.e., DIDanp-SIManp, DIDep-SIMep). Both brain areas are part of the DMN [33]. The precuneus is the area of the brain with the highest resting-state perfusion and with perfusion decreases during non-self-referential, goal-directed actions [67]. We therefore conclude that in contrast to the DID simulating controls, DID patients engaged as ANP and EP in self-referential mental activity in response to resting-state instructions.

In line with our second hypothesis, we found different patterns of resting-state perfusion for ANP and EP in the patients. Compared to EP, ANP showed more metabolism in the bilateral thalamus (DIDanp-DIDep), and right thalamus activity was higher in controls simulating EP than in authentic EP (SIMep-DIDep). However, controls simulating ANP also had more bilateral thalamus metabolism than controls simulating EP (SIManp-SIMep). Whereas relatively high right thalamus activity for ANP in DID patients may not be a DID-specific finding, our result parallels prior PTSD studies conducted under rest [68] or using script-driven symptom provocation paradigms [26], [69], [70]. Lanius et al. [26], [69], [70] have reported that Flashback/Reliving PTSD patients (i.e., subjects characterized with positive dissociative/EP-like symptoms) had not shown thalamic activation during the recall of traumatic memories while „dissociated“ PTSD subjects (i.e., subjects characterized with negative dissociative/ANP-like symptoms) did. Kim et al. [68] found a positive correlation between right thalamic blood flow following rest instructions and the severity of current re-experiencing symptoms in PTSD patients. In one of the first neurobiological models of dissociation in trauma surviviors, the thalamus was proposed to play a central role [71]. Sensory and arousal signals parallel in the thalamus which relays the transmission to target brain areas. Under condition of high arousal, this transmission is altered. Kim et al. [68] speculated that lowering of thalamic activity represents a withdrawal of attention from external sensory stimuli which may provoke re-experiencing symptoms. In consert with these findings, our findings suggest that as ANP, DID patients are more open to external sensory stimuli than as EP. Because of ANP's habitual tendency to be numb and depersonalized, they may not have been that alarmed by our instructions to relax, close their eyes, and stay immobile in a loud narrow space. As EP, however, these instructions may have reminded them of traumatizing circumstances. To cope with the situation, EP may have attempted to avoid attending to the subjectively threatening external cues, implying low right thalamus perfusion.

However, at the same time, they may have become self-aware, focused on internally alarming bodily and emotional cues, and prone to reactivate painful memories. Indeed, comparing EP to ANP in DID patients (DIDep-DIDanp), we found increased rCBF in the primary somatosensory cortex, in several motor-related brain areas, and in the DMPFC (see **Figure 1**). In a number of independent studies, self-referential action was associated with activity in the DMPFC [72], [73], [74]. Increased DMPFC activity in DID as EP has also been observed in a previous backward masking paradigm in reaction to potentially threatening stimuli [16]. We suggest that in DID patients compared to ANP, EP was attending more to his/her self-state and somatosensory sensations. Combining these findings, we interpret that focused on interoceptive, bodily-emotional cues, as EP, the patients were highly aware of being a body in a threatening situation. This awareness may have triggered a tendency to engage in defense motor reactions. In line with these findings and consistent with TSDP, DID patients specifically reported as EP that the lack of a more explicit task to focus on while laying in the scanner was threatening.

In line with our third hypothesis, comparisons of ANP and EP in controls yielded different neural reactivity patterns than comparisons of ANP and EP in DID patients. The actors reported that they used two major strategies to fulfill their simulation task: 1) imagining being another person and 2) trying to experience the other person's feelings. According to cognitive and social neuroscience, the first strategy can be described as visual mental imagery [75] and the second as empathizing [76]. Visual imagery elicits neural activity in visual areas [75], [77]. The increased perfusion in visual areas for controls simulating ANP compared to controls enacting EP (SIManp-SIMep) suggests that as ANP, actors particularly engaged in visual imagery. As the participants were requested to keep their eyes closed, activation in occipital areas cannot be explained by visual perception. The inverse contrast (SIMep-SIManp) revealed a higher perfusion in the anterior insula, pars triangularis of the inferior frontal gyrus, frontal operculum, and OFC, which are known to be neural underpinnings of empathy. There are different definitions of empathy in the literature. The second strategy for simulating ANP and EP mentioned above involved empathy in the sense of “Einfühlen,” that is “feeling into someone” [78], [79]. The anterior anterior insula is associated with empathy for pain [80], [81]. Pain can occur beyond nociception and can be generalized to mental suffering of any sort [82], such as laying in a scanner as a traumatized anxious (part of a) person. The pars triangularis and the frontal operculum are part of the mirror neuron system (MNS). The main function of the MNS pertains to simulation. For example, observing another person's actions increases the firing rate of neurons that are also active when we actually perform those actions ourselves [83]. Thus, the MNS is involved in understanding the actions and intentions of others [84], [85]. Neuroimaging studies in autism spectrum disorder patients [86] and healthy adults [87] also suggest that the MNS plays a pivotal role in empathy. Carr et al. [87] proposed that in concert with the anterior insula, the MNS is involved in grasping the emotional states of others by physically and emotionally feeling what it is like to engage in the observed action. The OFC has been found to be active in empathy tasks as well [88], [89], [90]. OFC functioning is critical for social cognition and socially appropriate behavior. Taken together, our data support the idea that DID-simulated controls engaged in envisioning and feeling of what one is not, that is, in simulating ANP and EP.

The follow-up study revealed the involvement of the temporal lobe in DID patients compared to non-simulating controls (DID-NS, DIDanp-NS, DIDep-NS; see **Table 5**) predominantly on the left hemisphere. This finding converges with other imaging and electrophysiological investigations of dissociation supporting the link between dissociation and temporal lobe functioning [46], [91], [92], [93], [94]. This association has also been proposed by a number of studies investigating the relationship between temporal lobe epilepsy and DID [95], [96], [97], [98]. The temporal lobe has been regarded as the generator of dissociative states [94], [96], [99]. One explanation is that the temporal lobe has strong anatomical connections to the limbic system (e.g., amygdala, involved in processing emotional responses and the hippocampus, a key structure for episodic memory) [96]. Emotional and mnemonic functions are disturbed in trauma stressor-related disorders such as dissociative disorders [100], [101], [102], [103]. Dissociative parts of the personality involve biopsychosocial subsystem that involve their own feelings and conceptions of who they are, what the world is like, and how they relate to that world. This selectivity could be associated in part with temporal lobe activity. This possibility is in accordance with a previous study showing that temporal lobe functioning may mediate “switches” between dissociative parts of the personality [104].

EP but not ANP in DID showed increased activity in the OFC and DMPFC compared to non-simulating healthy participants (DIDep-NS, see **Table 5**). Ventromedial parts of the frontal cortex are crucial for computing the affective value of an external sensory stimulus and for linking this perception to appropriate guidance of behavior [105]. Previous findings suggest that the right-sided sector of the ventromedial part of the frontal cortex, as observed in this study, is crucial for judging and remembering the aversive value of an external cue [106] and is sensitive to threat-related cues [107]. The dorsal parts of the MPFC mediate the evaluation and monitoring of negative emotions [108]. In a recent study, EP was also associated with overactivation of the DMPFC in response to covert trauma-related stimuli [16]. In line with TSDP, EP's increased DMPFC and OFC activation might be associated with EP's continuous tendency to mind threat and to be fixated on threat-related cues.

In the inverse contrasts (NS-DID, NS-DIDanp, NS-DIDep; see **Table 5**), non-simulating healthy individuals showed a tendency to elevated neural activity in posterior parietal regions, the MPFC, and hippocampus. This perfusion pattern observed for non-simulating healthy participants resembles the one subserving scene construction that includes a temporo-parietal-frontal network [109], [110], [111]. Scene construction describes episodic simulation or imagery of future and past events [109]. Scene construction and default mode activity share internally directed attention [110], and brain networks mediating future thinking and episodic memory overlap [110], [111], [112], [113], [114]. The medial temporal lobe system, which has long been considered to be uniquely involved in remembering the past and which was observed in non-simulating healthy controls as well (i.e., hippocampus), is also required to flexibly recombine details from the past in order to simulate future episodes [113], [114], [115]. The idea that non-simulating healthy participants were mentally travelling in time is further supported by elevated activity within the frontal polar cortex involved in future imagery and prospective thinking [112], [114], [116], [117], and increased perfusion in the extrastriate visual cortex associated with remembering past events [118].

Based on these interpretations and empirical foundations, our follow-up study suggest that under resting instructions as ANP and EP, DID patients activate left temporal regions more than mentally healthy controls who do not simulate ANP and EP. These controls may have been less involved in the task and scanner environment allowing them to let their mind wander to their personal past and imagined future. Of note: further analysis suggests that these results are not explained by a time or scanner effect (see Information S1: **Text S2 in Information S1**, **Table S1 in Information S1**, **Figure S1 in Information S1**, **Figure S2 in Information S1**, **Text S3 in Information S1**).

Merckelbach and co-workers observed a robust correlation between alterations in consciousness, some of which may reflect dissociative tendencies and a trait known as fantasy proneness in mentally healthy individuals [119], [120]. They argued that the correlation between self-reports of childhood abuse and these tendencies is mediated by fantasy proneness. Fantasy prone individuals would mix their fantasies with real memories and would be prone to produce pseudomemories of autobiographical traumatic events [11], [12]. Fantasy proneness is a trait defined as the deep involvement of fantasy and imagination [121]. However, fantasy in DID may be a means to cope with childhood traumatization (Nijenhuis and Reinders, see Information S1 in [14]), and DID patients are not high fantasy prone [14]. Moreover, as ANP and as EP, DID patients had less perfusion in brain areas involved in imagination than healthy controls who did not simulate ANP and EP in the present study. These findings are at odds with the idea that DID involves fantasy proneness and actual fantasizing.

The study has several limitations. First, our study may have lacked the power to detect potential perfusion differences. Although our sample is one of the largest samples included in an fMRI study of DID to date, it was still relatively small. This was due to the difficulty finding DID patients who are able to alternate between ANP and EP at request and to remain activated, particularly as EP, for a substantial period of time in a challenging fMRI environment. Further resting-state studies in DID are needed to confirm our findings. Second, patients who can perform these difficult actions are the ones who have been in treatment for at least several years. Because treatment of DID fosters integration of the different dissociative parts and traumatic memories, studies such as ours are prone to underestimate biopsychosocial differences between these subsystems of the personality in untreated individuals with DID. Another limitation of the study is that only two of our patients were free of medication. Medication washout is not feasible with DID patients. However, it is important to note that medication does not explain the observed differences between ANP and EP in DID. Whereas DID patients typically have considerable comorbidity, comorbidity does not explain the essence of the disorder, that is, the division of the personality manifesting in dissociative symptoms. For example, significant differences for the severity of somatoform dissociation in DID and other mental disorders remained after statistically controlling for the influence of comorbid symptoms [122]. Nevertheless, future studies will need to evaluate axis I and axis II comorbidity to disentangle dissociative symptoms and their patterns of neural activation from comorbid symptoms. Further, although we debriefed the presence of the identity state under investigation, we did not obtain biophysiological information that the intended ANP and EP were present during the measurements beyond these parts' self-report. Because ANP and EP had different psychophysiological reactions to reminders of traumatizing events [14], future studies best also include parameters such as heart rate or electrodermal activity. Furthermore, studies of dissociative parts in DID and other dissociative disorders should systematically assess their first-person experiences during the experiment. This will allow a comparison of third-person scientific data and the participants' first-person, phenomenal experiences, thus bridging the epistemic gap between body/brain and mind.

In conclusion, the present study is the first to show that two different prototypes of dissociative parts (i.e., ANP and EP subtype active defense) are associated with different patterns of brain activity following rest instructions in a challenging environment. The study also demonstrates for the first time that in this context and in contrast to DID simulating actors, particularly but not exclusively as EP, DID patients activated brain structures involved in self-consciousness. Following these instructions, neural activation associated with motivated role-playing of ANP and EP by mentally healthy controls was different from neural activation associated with being a genuine ANP and EP. The study adds to the evidence from supraliminal and subliminal neuroimaging studies of ANP and EP in DID [14], [15], [16], [21], [22] that suggestion, role-playing, and fantasy proneness do not explain the disorder. Our results also show that dissociative parts of the personality do not particularly activate brain structures associated with imagination. The findings are consistent with clinical observations of DID patients and with TSDP, but inconsistent with the sociocognitive model of DID.

## Acknowledgments

We would like to thank the colleagues of Prof. Jäncke's lab for their helpful comments and Franz Liem for his technical support. We are indebted to Ekaterina Weder and Eva Zimmermann for their collaboration as research clinicians. Special thanks go to the patients and their therapists for participating in the study.

## Supporting Information

Information S1 (PDF)
