# Supplementary material for: Dissociative Part-Dependent Resting-State Activity in Dissociative Identity Disorder: A Controlled fMRI Perfusion Study
Source: PLoS One. 2014 Jun 12;9(6):e98795. doi: 10.1371/journal.pone.0098795 (PMC4055615; doi:10.1371/journal.pone.0098795)
Supplement: Information S1 — (PDF) [file pone.0098795.s001.pdf]

Supporting Information S1

Dissociative part-dependent resting-state activity in dissociative identity disorder: A controlled fMRI perfusion study

Yolanda R. Schlumpf, MSc[a§], Antje A. T. S. Reinders, PhD[b,c], Ellert R. S. Nijenhuis, PhD[d], Roger Luechinger, PhD[e], Matthias J. P. Van Osch, PhD[f], Lutz Jäncke, PhD[a,g]

a Division of Neuropsychology, Institute of Psychology, University of Zurich, Switzerland
b Department of Neuroscience, University Medical Center Groningen, and BCN Neuroimaging Center, University of Groningen, Groningen, The Netherlands
c Department of Psychosis Studies, Institute of Psychiatry, King's College London, London, United Kingdom
d Top Referent Trauma Center Mental Health Care Drenthe, Assen, The Netherlands
e Institute for Biomedical Engineering, University and ETH Zurich, Switzerland
f Department of Radiology, C.J. Gorter Center for High-Field MRI, Leiden University Medical Center, Leiden, The Netherlands
g International Normal Aging and Plasticity Imaging Center, University of Zurich, Switzerland

Please address correspondence to:
y.schlumpf@psychologie.uzh.ch

This PDF file includes:

Text S1-3
Table S1
Figure S1-2
Supporting Reference List

Text S1: Image acquisition for the non-simulating control group

The MRI data of the non-simulating control group (NS) were obtained at the University Hospital of Zurich with a 3-T Philips Ingenia whole-body magnetic resonance imaging equipped with a 15-channel head coil. Ingenia and Achieva use an identical software. The pulse sequence varied slightly from the one used for the DID patient group (DID) on the Achieva system regarding 1) background suppression labeling (Ingenia: 1710 ms/2860 ms; Achieva: 1680 ms/2760 ms) and 2) TE (Ingenia: 14 ms; Achieva: 12 ms). The pulse sequence for the acquisition of the T1-weighted image was identical on both systems.

Text S2: Controlling for inter-scanner variability

Scanner-to-scanner variability of activation makes it difficult to say if group differences (i.e., DID-NS; NS-DID) in fMRI response patterns are genuine results or just reflect scanner differences. Previous studies have already addressed this issue in the context of multicenter studies involving hardware and software differences across sites [1,2,3]. These studies have shown that the major sources of variability of fMRI measurements are based on the different scanner manufacturers and the scanners' field strengths. As all participants have been measured on a 3-T Philips system, these major scanner effects can be ignored in the present study. It is important to note that in Tahmasebi et al. (2012), scanner effects could only explain 2% of the total variance of the fMRI response. This observation is in line with studies showing that the inter-subject variance involves a higher part of the total variance on fMRI measurements compared to between-scanner variance [2,4].

Nevertheless, scanner dependent changes regarding the SNR can have an impact on the brain activation patterns [3,5,6,7]. In the present follow-up study, we checked statistically if the perfusion differences between the patient group (DID), measured on the Achieva system, and the non-simulating control group (NS), measured on the Ingenia system, are confounded by SNR differences of the two systems. For this purpose, we followed the

approach of Friedman and colleagues, who used a particular type of SNR, i.e. the signal-to-fluctuation-noise-ratio (SFNR) per subject as a covariate of no interest in the statistical model [3]. Friedman et al. (2006) showed that this statistical adjustment significantly reduced scanner effects on the effect size of the MRI signal in a study including data measured on high- and low-field scanners from different vendors. For SFNR, the "signal" (S) is defined as the mean intensity in a region over time, whereas "fluctuation noise" (FN) is the standard deviation in this region of the same time series after a 2nd order polynominal detrending [3]. The SFNR is calculated on a voxel-wise basis as S divided by FN (S/FN).

For the present control analysis, SFNR images were calculated using an in-house programmed Matlab script. Following Friedman's et al. (2006) approach, the median SFNR per subject in GM was calculated over a GM mask obtained from the segmentation of the 3D T1 image by thresholding the GM probability images at 0.5 (note that this was the same GM mask described in the Method section and used in the context of correcting for biological variation in total rCBF). Only the GM signal was taken into account as Friedman et al. (2006) observed that SFNR estimates only including GM voxels are more effective compared to SFNR values based on WM.

The same two-sample t-tests (DID-NS, NS-DID; DIDanp-NS, NS-DIDanp; DIDep-NS, NS-DIDep) as described in the Method section were performed again with the median GM SFNR per subject included as covariate of no interest in the statistical model [3]. For the contrasts DID-NS and NS-DID, the SFNR values of ANP and EP per DID patient were averaged. Again, a cluster-size threshold of 10 voxels and p-values of $p<0.00167$ were applied. Results are listed in **Table S1** and discussed in **Text S3**. For a direct visual comparison, the statistical parametric maps of the t-tests performed with and without the SFNR adjustment are contrasted in "glass brain" renderings in the **Figure S1**.

As the SFNR is related to activation effects [3,8,9], the SFNR is typically used as a sensitivity measurement of imaging systems. Three t-tests were performed (SPSS) in order to check for significant differences in the SFNR obtained on the same scanner (i.e., Achieva:

DIDanp-DIDep) and on two different scanners (i.e., Achieva versus Ingenia: DIDanp-NS, DIDep-NS). DIDanp, DIDep, and NS were all treated as independent groups in order to have the same statistical power for all three tests. It might be speculated that inter-scanner differences in SFNR are higher compared to intra-scanner differences. P-values were Bonferroni corrected and set at $p<0.0167$, one-sided). Results are depicted in **Figure S2** and discussed in **Text S3**.

Table S1: Resting-state regional cerebral blood flow (rCBF) differences between DID patients and non-simulating controls. The median SFNR in GM per subject was entered as covariate of no interest in the statistical model.

			MNI coordinates[a]				
	Brain area	Side	*x*	*y*	*z*	k*E*	*T* value
DID-NS	Middle temporal gyrus (temporal pole)	L	-62	4	-22	242	5.96
	Inferior temporal gyrus (temporal pole)	L	-36	2	-40	160	5.24
	Middle temporal gyrus	L	-70	-32	-10	26	4.90
	OFC	L	-6	26	-18	278	4.39
	Superior frontal gyrus	L	-26	42	50	16	4.39
	Middle temporal gyrus (temporal pole)	R	68	0	-16	11	3.41
NS-DID	Supramarginal gyrus	L	-58	-42	44	264	6.29
	Occipital pole (extrastriate cortex)	L	-24	-98	2	421	*5.16
	Angular gyrus	L	-52	-60	30	168	4.64
	Lateral occipital cortex (middle division)	L	-40	84	12	76	4.54
	Inferior frontal gyrus (pars opercularis)	L	-50	18	30	28	4.40
	MPFC (frontopolar cortex)	R	4	54	-10	90	4.29
	Inferior frontal gyrus (pars triangularis)	R	46	42	-2	26	4.19
	Lateral occipital cortex (superior division)	L	-24	-76	32	62	4.11
	Supramarginal gyrus	R	62	-36	44	63	4.09
	Superior temporal gyrus	R	58	0	2	12	4.01
	Middle frontal gyrus	R	30	-4	48	36	3.90
	Lateral occipital cortex (middle division)	R	48	-86	4	22	3.89
	Lateral occipital cortex (superior division)	R	32	-84	34	21	3.81
DIDanp-NS	Middle temporal gyrus	L	-70	-32	-10	31	5.55
	Middle temporal gyrus	R	68	-2	-12	52	4.73
	Middle temporal gyrus (temporal pole)	L	-62	4	-22	134	4.70
	Inferior temporal gyrus (temporal pole)	L	-36	2	-42	84	4.43
	Superior temporal gyrus (temporal pole)	L	-50	14	-24	10	3.67
	OFC	L	-22	34	-16	12	3.66
NS-DIDanp	Middle frontal gyrus	R	30	-2	50	172	5.42
	Supramarginal gyrus	L	-58	-40	44	105	4.83
	Lateral occipital cortex (inferior division)	L	-52	-74	4	117	4.68
	Occipital pole (extrastriate cortex)	L	-24	-98	2	151	4.66
	Inferior frontal gyrus (pars opercularis)	L	-48	18	28	29	4.52
	Insula (posterior)	L	-44	-12	8	66	4.51
	Postcentral gyrus	L	-62	-16	34	48	4.39
	Superior parietal lobe	L	-30	-62	48	29	4.16
	Supramarginal gyrus	R	62	-46	38	78	4.12
	Lateral occipital cortex (superior division)	L	-20	-78	40	51	4.11
	Angular gyrus	L	-50	-62	30	136	4.09
	Middle frontal gyrus	L	-26	32	40	20	3.97
	Lateral occipital cortex (inferior division)	R	34	-80	6	31	3.96
	Lateral occipital cortex (superior division)	R	32	-84	34	18	3.77

DIDep-NS	Middle temporal gyrus (temporal pole)	L	-64	2	-20	270	6.50
	OFC	R	6	24	-26	232	4.86
	DMPFC	R	16	64	34	19	4.60
	Superior temporal gyrus	L	-70	-30	-10	16	4.38
	Inferior temporal gyrus	L	-62	-38	-24	29	4.22
	Superior frontal gyrus	L	-16	38	30	11	3.82
	Superior frontal gyrus	R	20	2	58	19	3.61
NS-DIDep	Supramarginal gyrus	L	-58	-42	46	291	6.59
	Lateral occipital cortex (middle division)	L	-40	-84	12	64	5.34
	Angular gyrus	L	-52	60	30	180	5.13
	Occipital pole (extrastriate cortex)	L	-24	-98	2	529	*4.95
	Insula (posterior)	L	-34	-22	14	63	4.57
	MPFC (frontopolar cortex)	R	4	56	-12	132	4.50
	Supramarginal gyrus	R	62	-36	44	82	4.46
	Inferior frontal gyrus (pars opercularis)	L	-50	18	32	21	4.21
	Lateral occipital cortex (superior division)	L	-24	-76	32	67	4.14
	Inferior temporal gyrus	R	46	-40	-22	14	3.97
	Inferior frontal gyrus (pars triangularis)	R	46	42	-2	17	3.93
	Lateral occipital cortex (middle division)	R	48	-86	4	27	3.82
	Hippocampus	R	32	-32	-8	11	3.63
	Lateral occipital cortex (superior division)	R	32	-82	36	10	3.55

R/L, left or right hemisphere; k*E*, cluster-size in voxels (one voxel is 2x2x2mm); DID, patient group; NS, non-simulating control group; DIDanp, ANP DID group; DIDep, EP DID group; OFC, orbitofrontal cortex; MPFC, medial prefrontal cortex; DMPFC, dorsomedial prefrontal cortex

[a] MNI coordinates (in mm) refer to the maximum of signal change in each region

[*] corrected for multiple comparisons using cluster-level statistics, $p \leq 0.05$

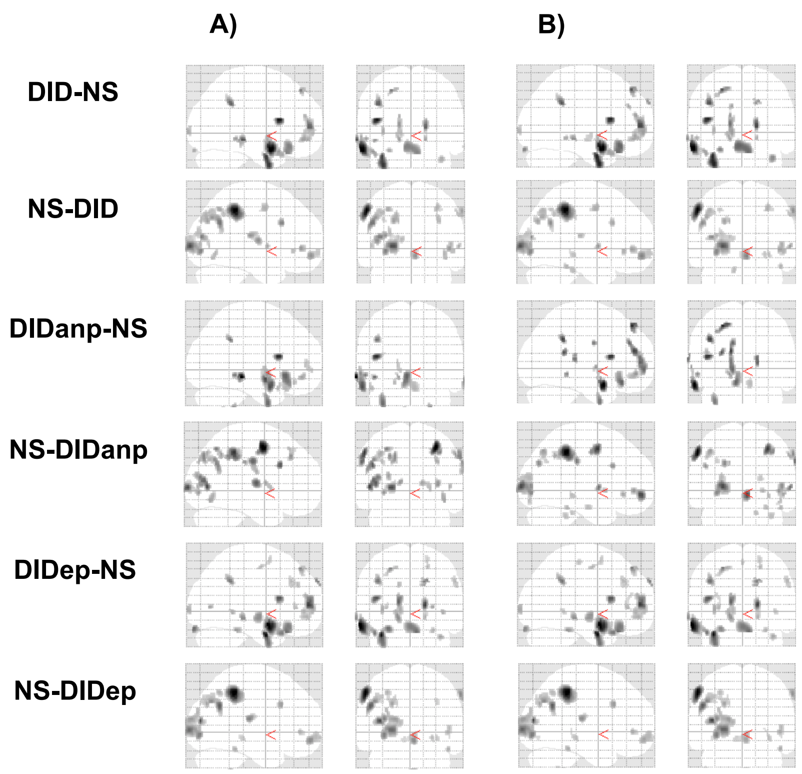

Figure S1: "Glass brain" renderings in saggital and coronal view comparing the statistical parametric maps for the t-tests A) with and B) without SFNR covariate adjustment. p<0.00167, cluster-size threshold = 10 voxels.

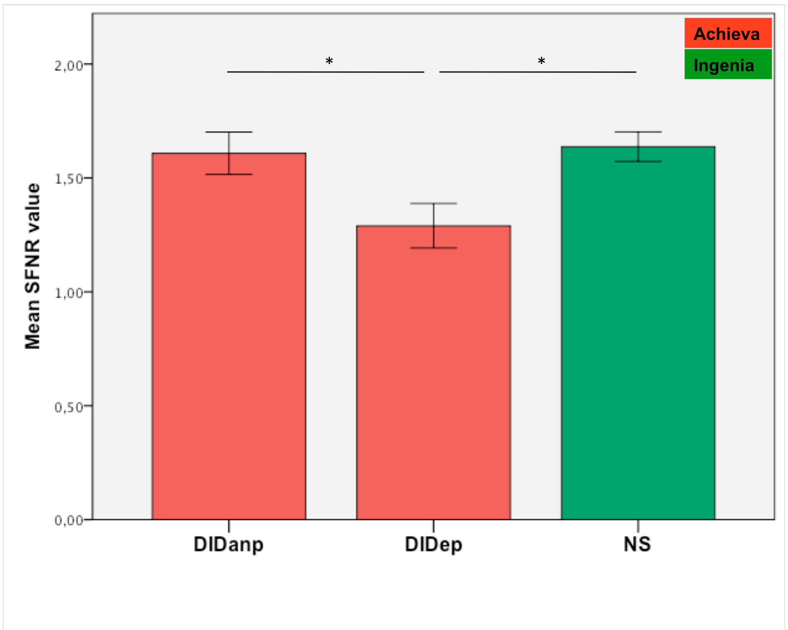

Figure S2: Within- and between scanner differences in SFNR. The figure depicts the mean SFNR value in GM in DIDanp and DIDep (measured on the Achieva system) and NS (measured on the Ingenia system) (±SD). *p<0.0167 (one-sided, Bonferroni corrected).

Text S3: Discussion of inter-scanner variability

SFNR estimates have been included as covariate of no interest in the statistical model to revoke potential scanner effects [3]. The results depicted in **Figure S1** indicate that our major group differences are not confounded by inter-scanner variability. The comparison of **Table S1** and **Table S4** (analysis not including measures of SFNR as covariate) shows that the left lateralized temporal activity in DID patients compared to NS (DID-NS; DIDanp-NS; DIDep-NS) and the prefrontal activity (i.e., OFC and DMPFC) in DIDep compared to NS (DIDep-NS) remains after adjustment with SFNR values. Slight differences could only be observed in NS compared to DID patients, as increased hippocampal activity was present in NS-DIDep, but vanished in NS-DID and NS-DIDanp. Nevertheless, in NS-DID, the cluster in the hippocampus just missed the cluster-size threshold due to one voxel (x=32, y=-32, z=8, $p<.00167$, k*E*= 9). The hippocampus has been described as a part of the scene construction network [10,11,12] in the Discussion section. Despite the slighly weaker hippocampal effect, we stick to the interpretation that NS engaged in imagination of future and past events, as in NS compared to DID patients, the remainder of the previously observed areas still showed increased perfusion after the statistical control with the SFNR values (see **Figure S1**), in particular the frontal polar cortex mediating future thinking [13,14,15,16] and the occipital cortex involved in imagining past events [17].

The results of the t-tests depicted in **Figure S2** are in line with the assumption that inter-scanner-differences are not the major source of the observed group differences. DIDep revealed the lowest SFNR, which might be explained by the largest movement artefacts in this subgroup. DIDanp was an adult dissociative part of the personality focused on functioning in daily life, whereas DIDep was a child-like anxious dissociative part fixed in traumatic memories [18]. Thus, DIDanp can be expected to be more similar to a healthy control person than DIDep. Insofar, significant differences in measures of SFNR values between DIDep and DIDanp and between DIDep and NS reflect inter-subject differences

more than inter-scanner differences (for the purpose of this comparison, DIDanp and DIDep are regarded as if they were different independent individuals). These interpretation accords previous findings showing that variability on fMRI measurements is mainly driven by the individual participant and not by technical changes [1,2,4].

Friedmann et al.'s (2006) findings demonstrating that inter-scanner variability can be reduced by controlling for SFNR differences is based on the blood-oxygenation-dependent (BOLD) signal. In perfusion based fMRI, the signal is prepared differently, but the read out is the same (echo-planar imaging (EPI) readout). Therefore, we regard it as appropriate to use the same approach as Friedman et al. (2006) did.

Taken together, after controlling for scanner variation in SFNR, the previous findings outlined in the Discussion section largely remain intact and the essence of our group differences does not change. Our data are in line with previous studies demonstrating that technical changes are not the major source of variability and support the feasibility of comparing data measured on identical scanner systems [1,2,4]

Supporting Reference List

1. Tahmasebi AM, Artiges E, Banaschewski T, Barker GJ, Bruehl R, et al. (2012) Creating probabilistic maps of the face network in the adolescent brain: a multicentre functional MRI study. Hum Brain Mapp 33: 938-957.
2. Zou KH, Greve DN, Wang M, Pieper SD, Warfield SK, et al. (2005) Reproducibility of functional MR imaging: preliminary results of prospective multi-institutional study performed by Biomedical Informatics Research Network. Radiology 237: 781-789.
3. Friedman L, Glover GH (2006) Reducing interscanner variability of activation in a multicenter fMRI study: controlling for signal-to-fluctuation-noise-ratio (SFNR) differences. Neuroimage 33: 471-481.
4. Costafreda SG, Brammer MJ, Vencio RZ, Mourao ML, Portela LA, et al. (2007) Multisite fMRI reproducibility of a motor task using identical MR systems. J Magn Reson Imaging 26: 1122-1126.
5. Kruger G, Glover GH (2001) Physiological noise in oxygenation-sensitive magnetic resonance imaging. Magn Reson Med 46: 631-637.
6. Kruger G, Kastrup A, Glover GH (2001) Neuroimaging at 1.5 T and 3.0 T: comparison of oxygenation-sensitive magnetic resonance imaging. Magn Reson Med 45: 595-604.
7. Triantafyllou C, Hoge RD, Krueger G, Wiggins CJ, Potthast A, et al. (2005) Comparison of physiological noise at 1.5 T, 3 T and 7 T and optimization of fMRI acquisition parameters. Neuroimage 26: 243-250.
8. Parrish TB, Gitelman DR, LaBar KS, Mesulam MM (2000) Impact of signal-to-noise on functional MRI. Magn Reson Med 44: 925-932.
9. Lowe MJ, Sorenson JA (1997) Spatially filtering functional magnetic resonance imaging data. Magn Reson Med 37: 723-729.
10. Hassabis D, Maguire EA (2007) Deconstructing episodic memory with construction. Trends Cogn Sci 11: 299-306.
11. Hassabis D, Maguire EA (2009) The construction system of the brain. Philos Trans R Soc Lond B Biol Sci 364: 1263-1271.
12. Schacter DL, Addis DR (2007) The cognitive neuroscience of constructive memory: remembering the past and imagining the future. Philos Trans R Soc Lond B Biol Sci 362: 773-786.
13. Addis DR, Wong AT, Schacter DL (2007) Remembering the past and imagining the future: common and distinct neural substrates during event construction and elaboration. Neuropsychologia 45: 1363-1377.
14. Okuda J, Fujii T, Ohtake H, Tsukiura T, Tanji K, et al. (2003) Thinking of the future and past: the roles of the frontal pole and the medial temporal lobes. Neuroimage 19: 1369-1380.
15. Okuda J, Fujii T, Yamadori A, Kawashima R, Tsukiura T, et al. (1998) Participation of the prefrontal cortices in prospective memory: evidence from a PET study in humans. Neurosci Lett 253: 127-130.
16. Burgess PW, Quayle A, Frith CD (2001) Brain regions involved in prospective memory as determined by positron emission tomography. Neuropsychologia 39: 545-555.
17. Addis DR, Schacter DL (2008) Constructive episodic simulation: temporal distance and detail of past and future events modulate hippocampal engagement. Hippocampus 18: 227-237.
18. Van der Hart O, Nijenhuis ERS, Steele K (2006) The Haunted Self: Structural Dissociation and the Treatment of Chronic Traumatization. New York: W.W. Norton & Company.
